# Supplementary material for: Forced changes in the Pacific Walker circulation over the past millennium
Source: Nature. 2023 Aug 23;622(7981):93–100. doi: 10.1038/s41586-023-06447-0 (PMC10550830; doi:10.1038/s41586-023-06447-0)
Supplement: Supplementary file 1 — This Supplementary Table describes records used in the ΔSLP reconstructions. For each record, the table outlines the: archive type, geographical location, temporal subset of the reconstructions to which the record contributed, Iso2k unique identifier (if sourced from the Iso2k database), seasonality (if stated by the original authors of the records), data source and primary reference. [file 41586_2023_6447_MOESM1_ESM.pdf]

nature portfolio

https://doi.org/10.1038/s41586-023-06447-0

Supplementary information

Forced changes in the Pacific Walker circulation over the past millennium

In the format provided by the authors and unedited

Supplementary Information for

Forced changes in the Pacific Walker Circulation over the past millennium

Georgina Falster et al.

Corresponding author: Georgina Falster, georgina.falster@anu.edu.au

The file includes:

Table S1

Table describing records used in the ΔSLP reconstructions

Archive	Latitude	Longitude	Temporal subset	Iso2k unique identifier	Seasonality	Source	Primary reference
Coral	13.60	144.84	1800	CO05ASGU01A	subannual	Iso2k	Asami et al. 2005[96]
Coral	-16.82	179.23	1800	CO05BAFI00A	annual	Iso2k	Bagnato et al. 2005[97]
Coral	17.93	-67	1800	CO08KIPR01A	annual	Iso2k	Kilbourne et al. 2008[98]
Coral	-15.94	166.04	1860	CO12GOVA01A	subannual	Iso2k	Gorman et al. 2012[99]
Coral	19.29	110.66	1860	CO13DESC01A	annual	Iso2k	Deng et al. 2013[100]
Coral	7.29	134.25	1800	CO14OSPA02A	subannual	Iso2k	Osborne et al. 2014[101]
Coral	-28.46	113.75	1860	CO14ZIHO01A	annual	Iso2k	Zinke et al. 2014[102]
Coral	-12.09	96.88	1860	CO18HECO01A	subannual	Iso2k	Hennekam et al. 2018[103]
GlacierIce	-73.59	-70.36	1860	IC08THGZ01A	annual	Iso2k	Thomas et al. 2009[104]
GlacierIce	-77.33	162.53	1200	IC11BEVL01A	annual	Iso2k	Bertler et al. 2011[105]
GlacierIce	-64.2	-57.69	1200	IC12MUJR01A	annual	Iso2k	Mulvaney et al. 2012[106]
GlacierIce	-77.52	167.68	1600	IC12RHME01A	annual	Iso2k	Rhodes et al. 2012[107]
GlacierIce	-79.38	-111.24	1800	IC13ST0101A	annual	Iso2k	Steig et al. 2013[108]
GlacierIce	-78.12	-95.65	1860	IC13ST1301A	annual	Iso2k	Steig et al. 2013[108]
GlacierIce	-77.06	-89.14	1800	IC13ST1501A	annual	Iso2k	Steig et al. 2013[108]
GlacierIce	-82	-110.01	1860	IC13ST2101A	annual	Iso2k	Steig et al. 2013[108]
GlacierIce	-86.5	-107.99	1600	IC13ST2401A	annual	Iso2k	Steig et al. 2013[108]
GlacierIce	-73.72	7.94	1800	IC13STN701A	annual	Iso2k	Steig et al. 2013[108]
GlacierIce	-74.57	-86.9	1800	IC13THFE01A	annual	Iso2k	Thomas et al. 2013[109]
GlacierIce	-13.93	-70.83	1200	IC13THQU01A	DJF	Iso2k	Thompson et al. 2013[110]
GlacierIce	-78.47	106.83	1800	IC14EKVK00A	annual	Iso2k	Ekaykin et al. 2014[111]
GlacierIce	77.45	-51.06	1800	IC15MANE00A	annual	Iso2k	Masson-Delmotte et al. 2015[112]
GlacierIce	-79.36	-161.7	1200	IC17BERI01A	annual	Iso2k	Bertler et al. 2018[113]
LakeSediment	38.34	34.46	1200	LS06JONA01	unknown	Iso2k	Jones et al. 2006[114]
Speleothem	16.21	-89.07	1400	SP12KEBZ01A	JJA	Iso2k	Kennett et al. 2012[115]
Speleothem	36.57	-118.78	1200	SP13MCUS01A	unknown	Iso2k	McCabe-Glynn et al. 2013[116]
Speleothem	-15.5	167	1600	SP13PAVU01A	unknown	Iso2k	Partin et al. 2013[117]
Wood	46.35	8.6	1800	TR07RECA01	MJ	Iso2k	Reynolds-Henne et al. 2007[118]
Wood	46.5	8.77	1800	TR07REVI01	MJ	Iso2k	Reynolds-Henne et al. 2007[118]
Wood	53.28	107.63	1800	TR08VOOL00	AM&JA	Iso2k	Tartakovsky et al. 2012[119]
Wood	-12.6	-69.2	1860	TR11BAPU02	Jan	Iso2k	Ballantyne et al. 2011[120]
Wood	30.31	91.52	1200	TR11GRTP00	Aug	Iso2k	Grießinger et al. 2011[121]
Wood	29.85	81.93	1800	TR11SAHU00	JJAS	Iso2k	Sano et al. 2012a[122]
Wood	19.9	101.2	1600	TR11XUPH01	MJJASO	Iso2k	Xu et al. 2013[123]
Wood	38.8	-105	1600	TR12BECO00	AMJJAS	Iso2k	Berkelhammer & Stott 2012[124]
Wood	21.67	104.1	1800	TR12SAMU01	MJJASO	Iso2k	Sano et al. 2012b[125]
Wood	68.4	-133.8	1800	TR13POMA00	AMJJ	Iso2k	Porter et al. 2014[126]
Wood	27.98	90	1800	TR13SAWA00	MJJAS	Iso2k	Sano et al. 2013[127]
Wood	54.56	-71.2	1200	TR15NACA00	JJA	Iso2k	Naulier et al. 2015[128]

Wood	52.22	-4.228	1860	TR15YOLL00	JJA	Iso2k	Young et al. 2015[129]
Wood	51.84	-4.15	1860	TR15YONW00	JJA	Iso2k	Young et al. 2015[129]
Wood	45.73	0.3	1400	TR16LAAN00	JJA	Iso2k	Labuhn et al. 2016[130]
Wood	48.38	2.67	1400	TR16LAFO00	JJA	Iso2k	Labuhn et al. 2016[130]
Wood	29.45	96.43	1600	TR16WEMI00	JJA	Iso2k	Wernicke et al. 2017[131]
Wood	30.42	95.07	1400	TR16WEXI00	JJA	Iso2k	Wernicke et al. 2017[131]
Wood	31.15	97.03	1200	TR17GRTP00	MJJAS	Iso2k	Grießinger et al. 2017[132]
Wood	32.22	77.22	1800	TR17SAMA00	JJAS	Iso2k	Sano et al. 2017[133]
Wood	-50.52	-70.12	1860	TR18GRPM00	ONDJ	Iso2k	Grießinger et al. 2018[134]
Wood	28.18	85.18	1860	TR18XUGA00	JJAS	Iso2k	Xu et al. 2018[135]
Wood	29.63	79.85	1800	TR18XUJA00	JJAS	Iso2k	Xu et al. 2018[135]
Speleothem	-22.1	114	1200	NA	annual	SISAL	Haig et al. 2014[136]
Speleothem	-17.2	144.6	1400	NA	annual	SISAL	Nott et al. 2007[137]
Speleothem	-9.49	159.97	1600	NA	subannual	SISAL	Maupin et al. 2014[138]
Speleothem	8.33	98.73	1800	NA	annual	SISAL	Tan et al. 2019[139]
Wood	19.3	97.97	1800	NA	MJJASO	Original publication	Pumijumnong et al. 2020[140]
Wood	34.48	110.08	1600	NA	annual	Original publication	Chen et al. 2016[141]
Wood	-11	-75	1860	NA	annual	Original publication	Humanes-Fuente et al. 2020[142]
Coral	-19.7	147.3	1800	NA	annual	Original publication	Lough 2007[143]
Coral	-18.9	146.6	1800	NA	annual	Original publication	Lough et al. 2015[144]

Table S1. All proxy records used to construct the ΔSLP reconstruction. For records sourced from the Iso2k database, information in the 'seasonality' column is from the 'isotopeInterpretation1_seasonality' metadata field, and data reference is from the 'pub1_citation' metadata field[23]. For all other records, seasonality information is inferred from the primary publication. For seasonality, 'unknown' refers to records where the authors of the primary dataset did not claim any particular seasonality for their record, but also did not explicitly state that the record represents an annually integrated signal. All records are annually-resolved, except those with 'subannual' in the seasonality column. Sub-annually resolved records were averaged over a calendar year. Archive names are as per the Iso2k database.
